# Pentraxin-3 Predicts Long-Term Cardiac Events in Patients with Chronic Heart Failure

**DOI:** 10.1155/2015/817615

**Published:** 2015-10-22

**Authors:** Haibo Liu, Xiaofang Guo, Kang Yao, Chunming Wang, Guozhong Chen, Wei Gao, Jie Yuan, Wangjun Yu, Junbo Ge

**Affiliations:** ^1^Shanghai Institute of Cardiovascular Diseases, Zhongshan Hospital, Fudan University, Shanghai 200032, China; ^2^Department of Cardiology, Yinzhou People's Hospital, Medical School of Ningbo University, Ningbo 315040, China; ^3^Clinical Laboratory Center, Yinzhou People's Hospital, Medical School of Ningbo University, Ningbo 315040, China

## Abstract

The aim of this study was to investigate the long-term prognostic value of pentraxin-3 (PTX3) in patients with chronic heart failure (CHF). 377 patients were prospectively followed up for 3 years to determine cardiac events including cardiac death or rehospitalization for worsening heart failure. The plasma PTX3 levels were significantly higher in CHF patients than in healthy subjects (*p* < 0.001), and they increased with advancing New York Heart Association (NYHA) Functional Classification (*p* < 0.001). Plasma PTX3 levels in CHF patients with cardiac events were significantly higher than in event-free patients (*p* < 0.001). We determined the normal upper limit of plasma PTX3 levels from the mean + 2 SD value of 64 control subjects (3.64 ng/mL). A Kaplan-Meier analysis revealed that patients with increased PTX3 (≥3.64 ng/mL) were at a higher risk for cardiac events than those without increased PTX3 (*p* < 0.01). A multifactorial Cox proportional hazards model showed that increased PTX3 (≥3.64 ngImL) was an independent risk factor for cardiac events in CHF patients (hazard ratio (HR) = 4.224, *p* < 0.01; 95% CI: 1.130–15.783). Plasma PTX3 levels are a long-term independent predictor of prognosis in patients with CHF.

## 1. Introduction

Chronic heart failure (CHF) is the end stage of many types of cardiovascular diseases, and the prognosis of patients with CHF is very poor. CHF is a leading cause of hospital admissions and death in developed countries [1], and CHF has also become a major cause of morbidity and mortality in developing countries. Thus, there is an urgent need for improvements in the risk stratification and prognosis of patients with CHF.

In recent years, studies have shown that inflammation is related to CHF and that plasma C-reactive protein (CRP) levels are associated with the prognosis of CHF patients [2–4]. Pentraxin-3 (PTX3) and CRP are members of the pentraxin family. CRP is a short pentraxin synthesized in the liver and may be an indicator of a systemic response to local inflammation [5, 6], while PTX3 is a long pentraxin produced mainly by dendritic cells, monocytes, fibroblasts, and vascular endothelial cells in response to primary inflammatory stimuli [7, 8], and this factor is highly expressed in the cardiovascular system [9–11]. PTX3 may reflect local inflammatory status in the cardiovascular system and thus may be a new biomarker of inflammation; however, this possibility will require more research.

We recently demonstrated that plasma levels of PTX3 were significantly higher in patients with stable coronary artery disease (CAD) after drug-eluting stent (DES) implantation and that these elevated levels of PTX3 were significantly and independently associated with the prevalence of major cardiovascular events (MACE) after DES implantation [12]. Moreover, a few recent studies also indicated that PTX3 levels were significantly higher in CHF patients and could potentially function as a predictor of adverse clinical outcomes in CHF patients [13–16].

All of these data were obtained in developed countries such as Japan and various European countries, and the causes of CHF in these countries differ from the causes of CHF in developing countries. To our knowledge, no data have been published about the association between PTX3 and CHF in developing countries. China is the largest developing country in the world; thus, data from China are likely to be valuable.

This study was designed to assess the efficacy of plasma PTX3 levels for the prediction of long-term cardiac events in CHF patients in China.

## 2. Materials and Methods

### 2.1. Patients

Between June 2010 and December 2011, we prospectively enrolled 406 patients with CHF who were admitted to Ningbo Yinzhou People's Hospital, Medical School of Ningbo University. Sixty-four age- and gender-matched healthy subjects from our medical examination center served as the control group for determining normal plasma levels of PTX3. These subjects were diagnosed as normal by physical examinations, chest X-ray, electrocardiogram, and echocardiography.

Two cardiovascular specialists confirmed all recruited CHF patients. The diagnostic criteria for patients with CHF included the following: (1) underlying heart disease; (2) dyspnea and edema of the lower limbs as well as other symptoms of heart failure; (3) abnormalities in at least one of the following objective indicators: etiological factors, the anatomy of the heart, and the cardiac function index (as assessed by chest X-ray or echocardiography). The inclusion criteria included the following: (1) impaired heart function (NYHA classes II to IV) on admission; (2) left ventricular ejection fraction (LVEF) ≤45%; and (3) impaired heart function (NYHA classes I and II) before discharge. Exclusion criteria included patients with renal dysfunction (serum creatinine >1.5 mg/dL), severe hepatic or lung disease, chronic or acute inflammation, and malignant disease. Patients who experienced myocardial infarction (MI) during the month prior to enrollment were also excluded.

This study was approved by the Ethics Committees of Ningbo Yinzhou People's Hospital, and all patients provided written informed consent.

In this study, demographic and clinical data, including age, gender, heart rate, diabetes mellitus, hypertension, hyperlipidemia, smoking, a previous history of myocardial infarction, NYHA class, and LVEF on admission, were collected from in-hospital medical records and patient interviews.

### 2.2. Venous Blood Samples and Laboratory Analyses

Venous blood samples were collected from patients on the second morning after admission under fasting conditions to measure levels of serum hsCRP and plasma PTX3. Whole blood was immediately collected into a tube containing ethylenediaminetetraacetate (EDTA) and then centrifuged at 2000 ×g for 15 min at room temperature; the plasma was kept frozen at −80°C until analysis. Plasma PTX3 concentrations were measured by enzyme-linked immunosorbent assay (ELISA; Perseus Proteomics Inc., Tokyo, Japan) as reported previously [17]. This assay can measure the plasma PTX3 concentration linearly between 0.1 and 20 ng/mL.

Normal PTX3 levels were determined based on the upper limit of plasma PTX3 levels from the mean + 2 SD value in control subjects.

### 2.3. Follow-Up and Endpoints

A total of 377 patients underwent clinical follow-up (follow-up rate, 92.9%) for three years after enrollment. The primary endpoint was cardiac events, which were defined as cardiac death or rehospitalization for worsening heart failure. A review of medical records and follow-up telephone interviews were conducted to survey for cardiac events among the enrolled patients. Reviews of the follow-up case notes were performed by two of the authors (Wang Chunming and Chen Guozhong) who were blinded to the PTX3 levels. All deaths were considered to be from cardiac causes unless an unequivocal noncardiac cause could be established.

### 2.4. Statistical Analysis

All data analyses were performed using SPSS, version 18.0 (SPSS, Chicago, IL, USA). Continuous data are expressed as the mean ± SD, and skewed variables are presented as the median value. Continuous variables were analyzed using unpaired Student's *t*-test or linear regression analysis; categorical variables were compared using the *χ* ^2^ test. If data were not normally distributed, the Mann Whitney *U*-test was used. Cox proportional hazard regression analysis was used to determine which variables were significantly related to cardiac events. Only variables with *p* values less than 0.05 in the univariate Cox regression analysis were entered into the multivariate Cox regression analysis. The hazard ratio (HR) and 95% confidence intervals (CIs) are presented. The log-rank test was performed to obtain the Kaplan-Meier probability estimates. A value of *p* < 0.05 was considered significant.

## 3. Results

### 3.1. Plasma PTX3 Levels in Control Subjects and CHF Patients

**Figure 1:**
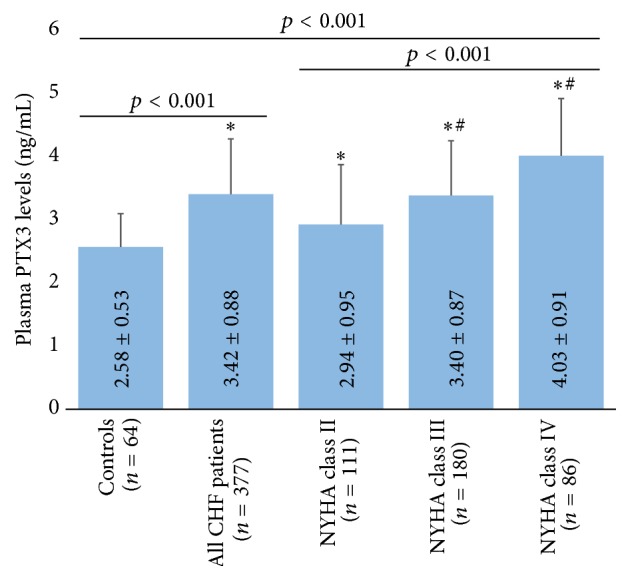
Plasma PTX3 levels in control subjects and CHF patients. ^*∗*^ *p* < 0.01 versus control, ^#^ *p* < 0.01 versus NYHA class II.

The plasma level of PTX3 in control subjects (37 males, 27 females, mean age: 76 ± 9 years) was 2.58 ± 0.53 ng/mL, but the corresponding value was 3.42 ± 0.88 ng/mL in the CHF patients (220 males, 157 females, mean age: 77 ± 9 years). As shown in Figure 1, the PTX3 levels were significantly higher in CHF patients than in control subjects (*p* < 0.01) and increased significantly with advancing NYHA functional class (*p* < 0.01).

### 3.2. Clinical Characteristics of CHF Patients with and without Cardiac Events

During the 3 years of follow-up, there were 152 (40.3%) cardiac events, including 54 (14.3%) cardiac deaths and 98 (26.0%) rehospitalization cases for worsening heart failure. Patients with a cardiac event had higher concentrations of PTX3, high-sensitivity C-reactive protein (hsCRP), and cardiac troponin (cTnI) (*p* < 0.001) compared with those without a cardiac event. Patients with a cardiac event had a higher prevalence of NYHA class >II and a higher heart rate and age (*p* < 0.001), but they showed decreased use of angiotensin converting enzyme inhibitor (ACE-I) or angiotensin receptor blocker (ARB) (*p* < 0.05) (Table 1).

### 3.3. Clinical Characteristics according to the Upper Limit Normal Plasma PTX3 Levels

The normal PTX3 levels were determined by the upper limit of plasma PTX3 levels (3.64 ng/mL) from the mean + 2 SD value in 64 control subjects (2.58 ± 0.53 ng/mL). Patients with increased PTX3 (≥3.64 ng/mL) had a higher prevalence of NYHA class >II (*p* = 0.039) as well as higher concentrations of hsCRP and cTnI (*p* < 0.001) compared to patients without increased PTX3 (Table 2).

### 3.4. Cardiac Events in CHF Patients

**Figure 2:**
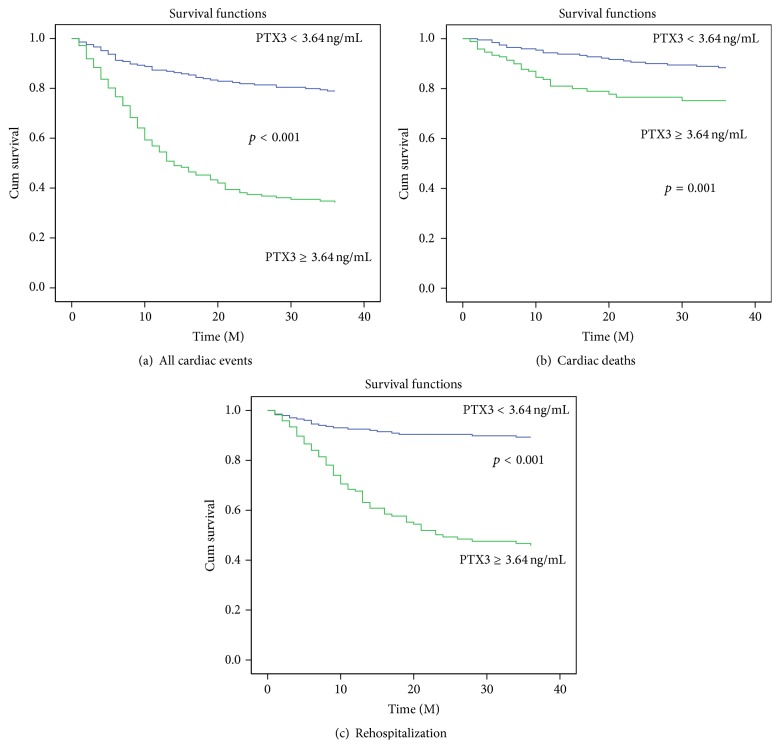
Kaplan-Meier analysis for all cardiac events (a), cardiac deaths (b), and rehospitalization (c) according to the upper limit normal plasma PTX3 levels (3.64 ng/mL). The *p* values were calculated using the log-rank test.

As shown in Table 3, the CHF patients with increased PTX3 levels had a higher prevalence of cardiac death (*p* < 0.05), rehospitalization for worsening HF (*p* < 0.001), and cardiac events (*p* < 0.001). Kaplan-Meier analysis also showed that the probability of cardiac events, cardiac death, and rehospitalization was significantly higher in the high-PTX3 group than in the low-PTX3 group (*p* < 0.001 or = 0.001, log-rank test) (Figure 2).

### 3.5. Univariate and Multivariate Cox Regression Analysis of Cardiac Events

PTX3, age, cTnI, NYHA class >II (*p* < 0.001), heart rate (*p* = 0.003), and hsCRP (*p* = 0.016) were significantly associated with cardiac events over the three-year period based on univariate Cox regression analysis (Table 4). A stepwise multivariate Cox regression analysis was performed that included age, heart rate, and prevalence of NYHA class >II, PTX3, cTnI, and hsCRP. PTX3 (hazard ratio (HR) 4.154, *p* = 0.005; 95% CI, 1.130–15.783), cTnI (HR 1.808, *p* = 0.008; 95% CI, 1.208–2.686), NYHA class >II (HR 3.018, *p* < 0.001; 95% CI, 1.818–4.998), and age (HR 2.518, *p* = 0.020; 95% CI, 1.030–6.158) were independently associated with cardiac events over the three-year follow-up period. hsCRP was not independently associated with cardiac events over that same time period (Table 4).

## 4. Discussion

In this study, we found that plasma PTX3 levels were significantly higher in CHF patients than in healthy subjects, that these levels increased with advancing NYHA functional classification, and that the plasma levels of PTX3 can significantly predict future cardiac events in CHF patients. Moreover, in a stepwise multivariate Cox regression analysis, which included well-known clinical and biochemical risk factors for CHF, the plasma levels of PTX3 remained an independent predictor of cardiac events in CHF patients. These findings suggest that PTX3 may be a reliable predictor for risk stratification in CHF patients, and measuring PTX3 may substantially improve the risk stratification of CHF patients.

In recent years, studies on the relationship between plasma PTX3 levels and CHF have made little progress. Suzuki et al. [16] found that PTX3 levels increased significantly in 196 CHF patients compared to 60 healthy controls and that they increased with advancing NYHA functional class. These authors also demonstrated that the plasma PTX3 level could be a prognostic risk factor in CHF patients. Similarly, Kotooka et al. [18] indicated that the plasma PTX3 levels were higher in 37 CHF patients with dilated cardiomyopathy than in healthy subjects. PTX3 levels might be a potentially useful biomarker for predicting prognosis as well as detecting inflammatory status in CHF patients. Recently, the CORONA and GISSI-HF trials showed that an elevated PTX3 concentration was associated with age and advanced NYHA class, and this marker independently predicted fatal outcomes in CHF patients over a 3-month period [14]. Further studies showed that high plasma PTX3 levels were correlated with future cardiovascular events in CHF patients with a normal ejection fraction [13, 15].

However, all of these data were obtained in developed countries, such as Japan and various European countries. To the best of our knowledge, we are the first to report an independent association between plasma levels of PTX3 and adverse cardiac events among CHF patients in a developing country. The risk stratification of CHF patients may be different between developed and developing countries because the causes of CHF and the extent of inflammatory activation are not the same.

In the present study, the normal plasma level of PTX3 from 64 healthy subjects was 2.58 ± 0.53 ng/mL, which is higher than that obtained in a large sample of healthy Japanese subjects [19]. This result might have occurred because our subjects were significantly older (76 ± 9 versus 60 ± 11 years), as plasma levels of PTX3 are known to increase with age [19]. However, the plasma PTX3 level in our CHF population was 3.42 ± 0.88 ng/mL, which is slightly lower than that reported by other studies in developed countries. Although the blood samples were drawn at a similar time, the average age in our study was older than that in the other studies [13, 14, 16]. This indicates that the increased inflammatory activation of CHF patients in China was not as significant as that observed in developed countries. There are no prior data on whether plasma PTX3 levels are associated with adverse cardiac events in China.

Our study demonstrated that the plasma level of PTX3 was not only risk factor but also an independent predictor of cardiac events in CHF patients. These results suggest that PTX3 could be considered as a universal risk factor in CHF patients.

In the present study, we indicated that both PTX3 and hsCRP were associated with cardiac events in CHF patients based on univariate Cox regression analysis; however, in the stepwise multivariate Cox regression analysis, only PTX3 was an independent predictor of adverse cardiac events in CHF patients. Our results are consistent with those of previous studies [13, 18], which suggest that PTX3 is a more reliable inflammatory predictor than CRP in CHF patients. Recent studies indicated that PTX3 was significantly related to hsCRP in CHF patients by correlation analysis [16, 18]. This association and the higher specificity of PTX3 for localized inflammation in the cardiovascular system [10, 11] might be responsible for the superior prognostic value of PTX3. In addition, our findings that age, NYHA class >II, and cTnI were also independently associated with cardiac events in CHF patients are consistent with the data in previous studies [20–22].

This study had several limitations. First, although this was a long-term prospective study, our data were derived from a relatively small number of patients in a single center in China; therefore, more studies must be performed in developing countries to confirm that PTX3 could be considered as a universal risk factor in CHF patients. Second, we only assessed the plasma PTX3 levels on the second morning after admission under fasting conditions. Serial measurements of PTX3 might be more useful for evaluating changes in inflammatory status in CHF patients and estimating risk during the follow-up period. Finally, our multifactorial Cox proportional hazards model did not include brain natriuretic peptide (BNP) or LVEF, which are strong predictors of CHF prognosis, due to insufficient data. Therefore, a multicenter trial with a large study population derived from different developing countries that includes BNP and LVEF data is necessary to validate the clinical importance of PTX3 in the future.

## 5. Conclusions

In conclusion, PTX3 could be considered as a universal risk factor and was more reliable than CRP in CHF patients. Measuring plasma PTX3 levels may substantially improve the risk stratification of CHF patients.

## Figures and Tables

**Table 1:** Clinical characteristics of patients with and without a cardiac event.

	All patients (*n* = 377)	Event-free (*n* = 225)	Cardiac event (*n* = 152)	*p* value
Age (years)	77.1 ± 9.1	75.0 ± 8.9	79.6 ± 9.3	<0.001
Males, *n* (%)	220 (58.5)	131 (58.2)	89 (58.6)	0.5175
Heart rate, beats/min	76 ± 18	74 ± 15	80 ± 18	<0.001
NYHA class >II, *n* (%)	266 (70.7)	134 (59.6)	129 (84.9)	<0.001
CHD, *n* (%)	196 (52.1)	114 (50.7)	82 (53.8)	0.599
Diabetes mellitus, *n* (%)	52 (13.8)	29 (12.9)	23 (15.1)	0.546
Hypertension, *n* (%)	143 (38.0)	89 (39.6)	54 (35.5)	0.450
Hyperlipidemia, *n* (%)	94 (25.0)	54 (24.0)	40 (26.3)	0.629
Current smoking, *n* (%)	115 (30.6)	68 (30.2)	47 (30.9)	0.910
PTX3 (ng/mL)	3.42 ± 0.88	3.088 ± 0.99	3.911 ± 0.83	<0.001
cTnI (ng/mL)	0.49 ± 0.24	0.24 ± 0.14	0.84 ± 0.45	<0.001
hsCRP (ng/mL)	6.72 ± 4.67	6.44 ± 4.35	7.22 ± 4.37	<0.001
Medical therapy				
Aspirin, *n* (%)	349 (92.8)	204 (90.6)	145 (95.4)	0.211
*β*-blockers, *n* (%)	254 (67.6)	160 (71.1)	94 (61.8)	0.073
ACE-I/ARB, *n* (%)	266 (70.7)	169 (75.1)	97 (63.8)	0.021
Diuretics, *n* (%)	344 (91.5)	204 (90.7)	140 (92.1)	0.712

**Table 2:** Clinical characteristics according to the upper limit normal plasma PTX3 levels^*∗*^ in CHF patients.

	PTX3 <3.64 ng/mL (*n* = 205)	PTX3 ≥3.64 ng/mL (*n* = 172)	*p* value
Age (years)	76.8 ± 9.5	77.5 ± 9.7	0.412
Males, *n* (%)	116 (56.6)	104 (60.5)	0.464
Heart rate, beats/min	75 ± 17	77 ± 20	0.346
NYHA class >II, *n* (%)	123 (60)	143 (83.1)	<0.001
CHD, *n* (%)	103 (50.2)	93 (54.1)	0.471
Diabetes mellitus *n* (%)	22 (10.7)	30 (17.4)	0.072
Hypertension, *n* (%)	83 (40.6)	60 (34.9)	0.287
Hyperlipidemia, *n* (%)	50 (24.4)	44 (25.6)	0.812
Current smoking, *n* (%)	60 (29.3)	55 (32.0)	0.577
cTnI (ng/mL)	0.34 ± 0.16	0.64 ± 0.28	<0.001
hsCRP (ng/mL)	5.27 ± 4.45	8.17 ± 5.43	<0.001
Medical therapy			
Aspirin, *n* (%)	189 (92.2)	160 (93.0)	0.845
*β*-blockers, *n* (%)	142 (69.3)	112 (65.1)	0.440
ACE-I/ARB, *n* (%)	151 (73.7)	115 (66.9)	0.174
Diuretics, *n* (%)	185 (90.2)	159 (92.4)	0.471

^*∗*^Normal PTX3 levels are determined based on the upper limit of plasma PTX3 levels from the mean ± 2 SD value in 64 control subjects (2.58 ± 0.53 ng/mL).

**Table 3:** Risk stratification of CHF patients based on increased PTX3 (above the upper limit normal plasma PTX3 level of 3.64 ng/mL).

	PTX3 <3.64 ng/mL	PTX3 ≥3.64 ng/mL	*p* value
	(*n* = 205)	(*n* = 172)	
Cardiac events	43	109	<0.001
Cardiac death	22	32	0.038
Rehospitalization for worsening HF	21	77	<0.001

**Table 4:** Univariate and multivariate Cox regression analysis of major adverse cardiovascular events.

	HR	95% CI	*p* value
Univariate analysis			
Age	2.609	1.371–4.992	<0.001
Males	0.760	0.534–1.082	0.128
Heart rate	1.189	1.058–1.328	0.003
NYHA class >II	3.493	2.089–5.848	<0.001
PTX3	4.399	3.081–6.281	<0.001
cTnI	1.591	1.296–1.954	<0.001
hsCRP	1.248	1.043–1.492	0.016
CHD	0.778	0.548–1.218	0.278
Diabetes mellitus	1.013	0.978–1.048	0.477
Hypertension	0.923	0.521–1.622	0.773
Hyperlipidemia	1.022	0.989–1.055	0.193
Current smoking	1.018	0.948–1.091	0.568
Aspirin	1.091	0.793–1.501	0.594
*β*-blockers	0.780	0.314–1.864	0.526
ACE-I/ARB	0.962	0.710–1.385	0.962
Diuretics	0.990	0.975–1.006	0.226
Multivariate analysis			
Age	2.518	1.030–6.158	0.020
PTX3	4.154	1.130–15.783	0.005
cTnI	1.808	1.208–2.686	0.008
NYHA class >II	3.018	1.818–4.998	<0.001

## References

[1] Stewart S., MacIntyre K., Hole D. J., Capewell S., McMurray J. J. V. (2001). More ‘malignant’ than cancer? Five-year survival following a first admission for heart failure. *European Journal of Heart Failure*.

[2] Lamblin N., Mouquet F., Hennache B. (2005). High-sensitivity C-reactive protein: potential adjunct for risk stratification in patients with stable congestive heart failure. *European Heart Journal*.

[3] Kardys I., Knetsch A. M., Bleumink G. S. (2006). C-reactive protein and risk of heart failure. The Rotterdam study. *American Heart Journal*.

[4] Williams E. S., Shah S. J., Ali S., Na B. Y., Schiller N. B., Whooley M. A. (2008). C-reactive protein, diastolic dysfunction, and risk of heart failure in patients with coronary disease: Heart and Soul Study. *European Journal of Heart Failure*.

[5] Pepys M. B., Hirschfield G. M. (2003). C-reactive protein: a critical update. *Journal of Clinical Investigation*.

[6] Agrawal A. (2005). CRP after 2004. *Molecular Immunology*.

[7] Garianda C., Hirsch E., Bozza S. (2002). Non-redundant role of the long pentraxin PTX3 in anti-fungal innate immune response. *Nature*.

[8] Breviario F., D'Aniello E. M., Golay J. (1992). Interleukin-1-inducible genes in endothelial cells. Cloning of a new gene related to C-reactive protein and serum amyloid P component. *Journal of Biological Chemistry*.

[9] Introna M., Vidal Alles V., Castellano M. (1996). Cloning of mouse ptx3, a new member of the pentraxin gene family expressed at extrahepatic sites. *Blood*.

[10] Rolph M. S., Zimmer S., Bottazzi B., Garlanda C., Mantovani A., Hansson G. K. (2002). Production of the long pentraxin PTX3 in advanced atherosclerotic plaques. *Arteriosclerosis, Thrombosis, and Vascular Biology*.

[11] Savchenko A. S., Imamura M., Ohashi R. (2008). Expression of pentraxin 3 (PTX3) in human atherosclerotic lesions. *Journal of Pathology*.

[12] Haibo L., Xiaofang G., Chunming W. (2014). Prognostic value of plasma pentraxin-3 levels in patients with stable coronary artery disease after drug-eluting stent implantation. *Mediators of Inflammation*.

[13] Matsubara J., Sugiyama S., Nozaki T. (2011). Pentraxin 3 is a new inflammatory marker correlated with left ventricular diastolic dysfunction and heart failure with normal ejection fraction. *Journal of the American College of Cardiology*.

[14] Latini R., Gullestad L., Masson S. (2012). Pentraxin-3 in chronic heart failure: the CORONA and GISSI-HF trials. *European Journal of Heart Failure*.

[15] Matsubara J., Sugiyama S., Nozaki T. (2014). Incremental prognostic significance of the elevated levels of pentraxin 3 in patients with heart failure with normal left ventricular ejection fraction. *Journal of the American Heart Association*.

[16] Suzuki S., Takeishi Y., Niizeki T. (2008). Pentraxin 3, a new marker for vascular inflammation, predicts adverse clinical outcomes in patients with heart failure. *American Heart Journal*.

[17] Inoue K., Sugiyama A., Reid P. C. (2007). Establishment of a high sensitivity plasma assay for human pentraxin3 as a marker for unstable angina pectoris. *Arteriosclerosis, Thrombosis, and Vascular Biology*.

[18] Kotooka N., Inoue T., Aoki S., Anan M., Komoda H., Node K. (2008). Prognostic value of pentraxin 3 in patients with chronic heart failure. *International Journal of Cardiology*.

[19] Yamasaki K., Kurimura M., Kasai T., Sagara M., Kodama T., Inoue K. (2009). Determination of physiological plasma pentraxin 3 (PTX3) levels in healthy populations. *Clinical Chemistry and Laboratory Medicine*.

[20] Beygui F., Anguita M., Tebbe U. (2015). A real-world perspective on the prevalence and treatment of heart failure with a reduced ejection fraction but no specific or only mild symptoms. *Heart Failure Reviews*.

[21] Horwich T. B., Patel J., MacLellan W. R., Fonarow G. C. (2003). Cardiac troponin I is associated with impaired hemodynamics, progressive left ventricular dysfunction, and increased mortality rates in advanced heart failure. *Circulation*.

[22] Tsutamoto T., Kawahara C., Nishiyama K. (2010). Prognostic role of highly sensitive cardiac troponin I in patients with systolic heart failure. *American Heart Journal*.

